# Ethics of alternative trial designs and methods in low-resource settings

**DOI:** 10.1186/s13063-019-3841-2

**Published:** 2019-12-19

**Authors:** Rieke van der Graaf, Phaik Yeong Cheah

**Affiliations:** 1 University Medical Center Utrecht, Julius Center for Health Sciences and Primary Care, Utrecht, Netherlands; 2 Mahidol Oxford Tropical Medicine Research Unit, Faculty of Tropical Medicine, Mahidol University, Bangkok, Thailand; 3 Centre for Tropical Medicine and Global Health, Nuffield Department of Medicine, University of Oxford, Oxford, UK; 4 The Ethox Centre, Nuffield Department of Population Health, University of Oxford, Oxford, UK

**Keywords:** Alternative trial designs, Cluster randomized trials, Ethics, Adaptive trials, Controlled human challenge studies, Stepped wedge cluster randomized trials

## Abstract

This editorial introduces articles in this Special Issue, which are based on presentations given at the 2017 meeting of the Global Forum of Bioethics in Research meeting. The main themes presented at the meeting were the use of cluster randomized trials, stepped-wedge cluster randomized trials, and controlled human infection models in research conducted in low-resource settings. The editorial sets out which ethical issues may arise in the context of alternative trial designs and describes the articles in this issue that addresses some or more of the ethical issues, such as justification of the research design, risk-benefit evaluations and consent.

## Background

In health research, the randomized controlled trial (RCT) has traditionally been the gold standard for evaluating the efficacy and safety of new interventions. However, conventional RCTs are notorious for being time-consuming, having high costs, and not resembling real-world populations. Moreover, there are methodological reasons for choosing alternative study designs over conventional RCTs. For example, cluster randomized trials (CRTs) are essential when the intervention being evaluated is delivered at the cluster level (such as public health interventions) or at the health professional level (such as knowledge translation or health systems interventions). In some cases the cluster randomized design is the only option. For example, vaccine trials that seek to measure herd immunity, as well as trials involving mass drug administrations (MDAs), must be cluster randomized, since the effect has to be assessed at the cluster level rather than the individual level. A recent example of the latter is an MDA study of antimalarial drugs in Myanmar, Vietnam, Cambodia, and Laos [1]. Sixteen villages across these four countries were randomized to either MDA or control. In the MDA arm, entire villages received MDA with basic malaria control tools such as distribution of insecticide-treated bednets, early diagnosis, and treatment. The control villages received basic malaria control tools. The justification for using a CRT instead of a conventional RCT was that the MDA intervention could only be administered at the village level: antimalarials were given to everyone in the MDA village including those who were not ill but who may have harbored malaria parasites. Asymptomatic individuals had to be provided with the intervention (antimalarials), since these individuals act as a reservoir for malaria and a source of infection to others [2].

CRTs and stepped wedge CRTs (SW-CRTs) are nowadays common in low-resource settings, especially in the evaluation of public health interventions. SW-CRTs are a form of CRTs where not half of the clusters but all clusters will eventually receive the experimental intervention. In SW-CRTs an intervention is rolled out in a stepped manner at predefined intervals until eventually all clusters have received the intervention. SW-CRTs are attractive when there is the belief that the intervention will do more good than harm [3]. The potential to combine preferences for the intervention with implementation research renders SW-CRTs highly attractive for low-resource settings. For instance, ethical and practical reasons were the main rationale for using a SW-CRT in 2008 and 2009 when researchers examined “the effect of training residents in interpersonal and communication skills on women’s satisfaction with the doctor–woman relationship in labour and delivery rooms” [4]. The trial enabled the training of all 137 residents at four public maternity hospitals in Damascus, Syria and its surroundings, “thereby complementing their medical training in communication skills that is missing from their curriculum” [4].

Apart from alternative *designs,* an alternative *methodology* can be used to speed up drug or vaccine development, such as in controlled human infection models (CHIMs). These models involve deliberate exposure of healthy volunteers to an infection in a controlled setting with the aim of testing whether a drug or vaccine works. It can give researchers an indication that a drug or vaccine is safe and effective far more quickly than would be possible through large-scale population-based trials. CHIMs are regarded as promising methods in global health research, although there have only been few in low-resource settings in individuals drawn from populations at risk of the disease under study. CHIMs conducted in endemic settings produce results more relevant to the real-world planned use of the intervention [5–7]. At the same time, CHIMS cannot demonstrate the effectiveness of medications; this still necessitates large field trials. For example, the development of a dengue vaccine relies heavily on CHIM studies to select potential vaccine candidates before large-scale clinical trials are undertaken [8]. However, the results obtained by means of these dengue CHIM models still need further testing in conventional phase III RCTs [8].

Although there is increasing interest in the use of alternative designs and methods in both high- and low-resource settings, there is a paucity of literature discussing the ethical issues, in particular in the context of low-resource settings.

## Guidelines on alternative trial designs and methods

There are few specific ethical guidance documents for the use of alternative designs and methods. The use of alternative trial designs and methods is not part of the Declaration of Helsinki (2013) [9], although the main principles for human subjects research do apply. The only specific guidance document is the Ottawa Statement on the Ethical Design and Conduct of Cluster Randomized Trials (2012) [10]. It also influenced the 2016 International Ethical Guidelines for Health-related Research Involving Humans of the Council for International Organizations of Medical Sciences (CIOMS), which has a specific paragraph on the ethics of CRTs [11]. At the same time, several ethical issues have been highlighted in the literature that merit specific consideration when using these designs, such as delaying the roll-out of an intervention that is believed to be superior to the standard of care in SW-RCTs [3] and also the level of acceptable research risks in CHIMs when healthy participants are deliberately exposed to infectious agents [12]. The paucity of ethical reflections on the use of alternative designs and methods in low-resource settings beyond the context of public health emergencies combined with the increased attention for these designs justifies a Special Issue.

## Ethical issues

Researchers grapple with multiple ethical issues when considering alternative trial designs and methodologies. The manuscripts in this Special Issue reveal several cross-cutting themes. First, what is the scientific justification for deviating from a conventional trial? Will an alternative trial design answer the research question without compromising the scientific validity of the trial? For both CRTs and SW-CRTs, researchers have to ask whether practical or logistical reasons fully justify the choice of the research design. Cluster randomized trials (including SW-CRTs) are less efficient, more prone to bias, and expose more people to harm than conventional RCTs. Practical and logistical reasons for using a CRT or SW-CRT should be weighed against the possible advantages of more rigorous methods such as conventional RCTs.

Risk-benefit evaluations represent a second cross-cutting theme. As Spencer Hey and colleagues pointed out last year, “new trial designs present challenges for applying equipoise and discussing risks with patients and participants” [13]. For example, SW-CRT trial designs delay the roll-out of the intervention to some of the control groups. Since one reason to use an SW-CRT instead of a conventional CRT might be to study the roll-out of an intervention that is “believed” to be superior to the standard of care and hence should not be withheld from the control group, the risks of delaying the roll-out should be taken into account [8]. In CHIM studies, risk-benefit evaluation can also be highly challenging [12]. In CHIM studies, interventions are tested on healthy volunteers. What should be the absolute upper risk limits (if any) in research with competent consenting participants who do not stand to benefit clinically from the research? CHIM participants take risks, but there is usually little or no prospect of individual clinical benefit. That said, volunteers from resource-poor communities in endemic areas may stand to gain more personally or for their communities than volunteers in the resource-rich, non-endemic areas where CHIMs are more usually conducted.

The third common ethical challenge encountered in all alternative designs discussed in this Special Issue is that of consent. Empirical studies have suggested that most clinical trial participants do not understand complex clinical trial terminology and concepts such as randomization [14, 15]. In CRTs and SW-CRTs, refusal of consent by individuals in a cluster may be meaningless when it is virtually impossible to opt out o receiving the intervention. For instance, in a health systems intervention, patients may have no option to refuse participation, as individuals cannot always move to another location to receive an alternative form of care. Researchers also grapple with obtaining participant consent for CHIM studies. A study in Kenya showed that of 143 potential participants screened for their understanding of the proposed CHIM study, 100% required at least two attempts and 55% required three attempts to answer correctly all questions on a questionnaire designed to test their understanding [6].

These and other issues were discussed at the 2017 Global Forum on Bioethics in Research (GFBR) meeting, which took place in Bangkok, Thailand, on 28 and 29 November 2017. The GFBR, supported by the Wellcome Trust, the Bill & Melinda Gates Foundation, the National Institutes of Health, and the UK Medical Research Council, hosts annual meetings on contemporary bioethics topics, such as research with pregnant women (2016) [16] and data sharing and biobanking (2018). The 2017 meeting focused on the ethics of alternative trial designs, namely adaptive trials, CRTs, including SW-CRTs, and CHIMs in low- and middle-income country research. Some do not consider the designs discussed in this Special Issue as ”alternative,” but for practical reasons, we will use this term throughout this Special Issue [17].

## Aims of this Special Issue

The articles in this Special Issue are based on presentations given at the 2017 GFBR (http://www.gfbr.global/) meeting. This issue follows the main themes presented at the 2-day meeting: CRTs, SW-CRTs, and CHIMs. All presenters in the thematic sessions were invited to contribute to this Special Issue. Chocko et al. discuss the use of CRTs in two case studies, a pragmatic open-labeled CRT of a drug to prevent cardiovascular disease conducted within the Golestan Cohort Study in Iran and an adaptive CRT to investigate the effect of interventions to increase uptake of HIV testing and linkage to care or prevention among male partners of pregnant women in Malawi [18]. Joag et al. present an ethical evaluation of SW-CRT use in the Que Vivan Las Madres study conducted in two Guatemalan districts and the Atmiyata study conducted in Gujarat India [19]. Raymond et al. and Palacios and Shah discuss the use of CHIMs. Palacios and Shah discuss the ethical justifications for conducting Zika human challenge trials in endemic settings [20], while Raymond et al. describe the ethical and practical challenges when conducting *Salmonella* CHIMs in high- and low-resource settings [21]. Finally Hunt, Saenz, and Littler summarize the GFBR 2017 meeting [17]. Adaptive (platform) trials were also discussed at the meeting but did not result in additional papers, since one of the case studies, an Ebola trial in Sierra Leone, has been published elsewhere [22], and the other case study did not take place due to funding challenges.

The case studies in this Special Issue illustrate concrete ethical and practical issues related to alternative trial designs and methods experienced by the research community. We hope that they stimulate discussions among researchers and ethics committee members on the ethical and practical aspects of alternative trial designs and methods in both high- and low-income settings. We also urge researchers to include these designs in their toolkit of research methods, and to build the capacity of researchers to design and conduct such studies and ethics committees to review them. Finally, we encourage the research community to take into account existing ethics and regulatory requirements. At the same time, considering specific guidance for the use of these designs can improve conditions under which these designs and methods are being used and hence the protection of research participants.

## Data Availability

Not applicable.

## Abbreviations

CHIM: Controlled human infection model
CIOMS: Council for International Organizations of Medical Sciences
CRT: Cluster randomized trial
GFBR: Global Forum on Bioethics in Research
RCT: Randomized controlled trial
REC: Research Ethics Committee
SW-CRT: Stepped wedge cluster randomized trial
